# Precision wearable accelerometer contact microphones for longitudinal monitoring of mechano-acoustic cardiopulmonary signals

**DOI:** 10.1038/s41746-020-0225-7

**Published:** 2020-02-12

**Authors:** Pranav Gupta, Mohammad J. Moghimi, Yaesuk Jeong, Divya Gupta, Omer T. Inan, Farrokh Ayazi

**Affiliations:** 1 Georgia Institute of Technology, Atlanta, GA 30308 USA; 2 Department of Medicine, Emory University, Atlanta, GA 30308 USA

**Keywords:** Electrical and electronic engineering, Health care, Biomedical engineering

## Abstract

Mechano-acoustic signals emanating from the heart and lungs contain valuable information about the cardiopulmonary system. Unobtrusive wearable sensors capable of monitoring these signals longitudinally can detect early pathological signatures and titrate care accordingly. Here, we present a wearable, hermetically-sealed high-precision vibration sensor that combines the characteristics of an accelerometer and a contact microphone to acquire wideband mechano-acoustic physiological signals, and enable simultaneous monitoring of multiple health factors associated with the cardiopulmonary system including heart and respiratory rate, heart sounds, lung sounds, and body motion and position of an individual. The encapsulated accelerometer contact microphone (ACM) utilizes nano-gap transducers to achieve extraordinary sensitivity in a wide bandwidth (DC-12 kHz) with high dynamic range. The sensors were used to obtain health factors of six control subjects with varying body mass index, and their feasibility in detection of weak mechano-acoustic signals such as pathological heart sounds and shallow breathing patterns is evaluated on patients with preexisting conditions.

## Introduction

Congestive heart failure (CHF), a progressive condition wherein the heart is unable to adequately pump blood to meet the metabolic demands of the body, is a leading cause of hospitalization and mortality, and substantially degrades the quality of life of affected individuals.^1–3^ CHF impacts over 26 million people globally and the incidence is rapidly growing with the aging population demographics.^2,4^ One effective strategy to improve survival rate is to detect early presymptomatic pathophysiological changes associated with CHF and implement treatments proactively to slow its progression.^5–8^ However, early CHF is highly underdiagnosed and general screening of the population remains a major challenge.^9^

A promising approach for early diagnosis of CHF is to continuously monitor the mechano-acoustic cardiopulmonary signals over extended periods of time using wearable sensing systems, which are non-invasive, simple, and cost-effective.^10–15^ The signals convey physiological parameters that are essential for a comprehensive evaluation of the health status of individuals.^16,17^ For instance, the opening and closing of the heart valves, along with pumping of blood into the arteries and veins, produce acoustic signals in the frequency range of 20–1000 Hz.^18,19^ The existence of pathological sounds in these signals can indicate structural defects in the heart valves, blockage in arteries or ventricular dysfunction.^20,21^ In addition, micro-vibrations of the chest wall in the low frequency range of DC-100 Hz are linked to mechanical cardiac events and contain information about valve functionalities and cardiac muscle contractility.^22,23^ Moreover, changes in the lung volume during the respiratory cycle cause macro-motions of the thorax and abdomen with ultralow frequency below 1 Hz.^24^ This signal is useful to extract the respiratory pattern, which is significant in predicting clinical deterioration of patients with chronic airflow obstruction,^25,26^ and diagnose several other respiratory diseases.^27^

These mechano-acoustic signals comprise a wide range of intensities and frequencies; requiring, ultrasensitive sensors with high dynamic range and wide bandwidth to record these signals with high fidelity. In addition, these sensors must be small and lightweight to be easily accommodated in a wearable platform. Such a platform can enable continuous monitoring of the cardiopulmonary system over an extended period of time and facilitates early detection and comprehensive diagnosis.^12–14,28^ Existing systems including digital stethoscopes and accelerometers are not well-suited for low-profile, wearable, and high-precision monitoring systems. Digital stethoscopes rely on large size membranes to acquire weak acoustic signals emanating from the body. Miniature accelerometers with low-bandwidth have been used in limited capacity to obtain mechanical vibrations and record seismocardiogram (SCG) signals and respiratory patterns^29,30^ but fail to detect characteristic acoustic signatures of ventricular dysfunction such as pathological S_3_ heart sounds occurring in patients with congestive heart failure, and adventitious pulmonary sounds associated with deteriorating cardiopulmonary conditions.^17,21,31^ While preliminary studies using accelerometers in implantable defibrillators has been reported for monitoring cardio-mechanical signals in heart failure patients,^32^ these devices are unsuitable for preventive care as they require expensive and complex surgical procedures for implantation. Therefore, a wearable high-precision sensing system that can detect the sounds, signs, and symptoms and enable longitudinal monitoring of cardiopulmonary health, is needed to potentially address the delay in detection and improve accuracy of diagnosis.

Here, we present a wearable hermetically-sealed contact microphone to capture the body’s mechano-acoustic signals in a wide frequency range of DC-12 kHz and enable longitudinal study and monitoring of the cardiopulmonary system. The 2 × 2 mm encapsulated microsensor chip can record a wide range of vibrations on human skin, ranging from very low frequency (below 1 Hz) movements associated with the chest wall and body position to high frequency acoustic signals (up to 12 kHz) emanating from the heart and lungs. In comparison to existing accelerometers, the high sensitivity to micro-gravity level accelerations over a wide frequency range enables the application of our device in body motion tracking, SCG monitoring, recording pulmonary sounds occurring at high frequencies, as well as capturing weak abnormal heart sounds which require high-precision sensing. Additionally, the sensors benefit from wide dynamic range and operate from 10 µg to 16 g with a linear response. This allows the device to be used reliably under ambulatory conditions, without suffering from output saturation that may occur at high acceleration levels during body motion. Using this sensor, we have been able to detect pathological S_*3*_ heart sounds in patients with preexisting conditions while simultaneously measuring shallow breathing patterns. The devices are fabricated using a unique and high-precision fabrication technique, the high aspect-ratio combined poly- and single-crystal silicon micromachining technology (HARPSS),^33,34^ to achieve ultrasensitive, wideband and triaxial capacitive vibration transducer. The sensor with ultrahigh aspect-ratio gaps of greater than 150, which cannot be fabricated with conventional fabrication methods, simultaneously benefits from functionalities of contact microphones and triaxial accelerometers to enable recording cardiopulmonary sounds, heart rate (using SCG^22^ signals), respiration rate and physical activities of an individual. The performance of these hermetically-encapsulated sensors is not compromised by body sweat and environmental effects. Moreover, the sensor only responds to vibrations on the surface and is not susceptible to airborne acoustic noise in the environment. These features allow us to simultaneously monitor and evaluate multiple health factors of an individual and can assist in creation of new digital biomarkers for improved diagnostic evaluation. These sensors were initially tested on healthy subjects to record key health parameters such as phonocardiogram along with heart and respiration rate wherein they exhibited highly correlated metrics with medical-grade electronic stethoscope and electrocardiogram (ECG) signals. Subsequently, we also demonstrate their extraordinary sensitivity, with a small pilot study, by capturing weak mechano-acoustic signals such as pathological *S*_*3*_ heart sounds and shallow breathing patterns in patients with preexisting conditions.

## Results

### Mechanism of sensing health factors

**Fig. 1:**
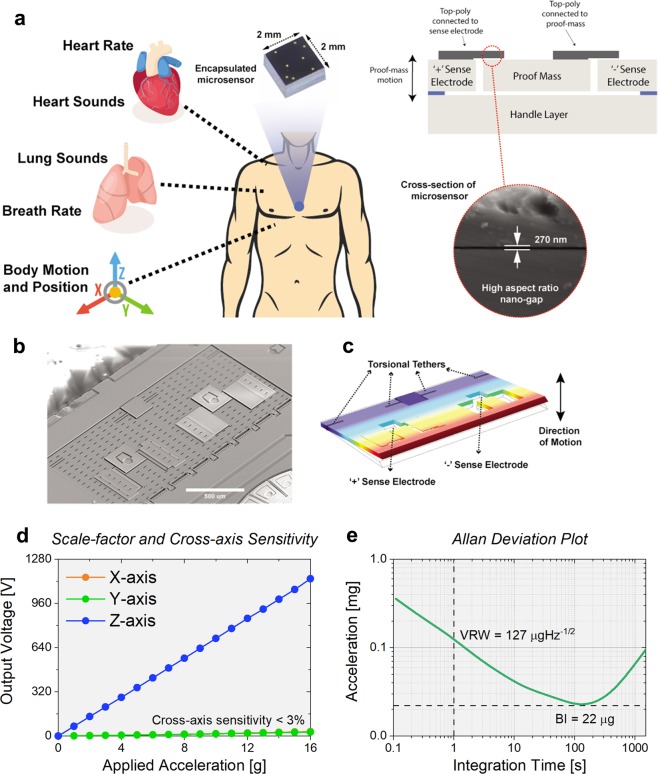
Hermetically-sealed sensor with nanogaps for cardiopulmonary health monitoring. **a** conceptual representation of encapsulated sensor positioned on the on the chest wall (blue circle) to simultaneously monitor heart rate, heart sounds, respiratory rate, breath sounds along with body motion and position. The exploded view displays the fabricated microsensor (2 × 2 × 1 mm) and its cross-sectional view showcasing enabling technology of high aspect-ratio (>150), ultra-thin 270 nm capacitive gap, **b** SEM of uncapped accelerometer contact microphone (ACM) device. The proof mass anchored on the side using torsional tethers **c** COMSOL Multiphysics simulation illustrating the operational mode shape of the sensor and showcasing the location of torsional tethers and sense electrodes **d** transducer response to normally applied acceleration with measured sensitivity of 76 mV/g and cross-axis sensitivity lower than 3%, **e** Allan deviation plot exhibiting low-noise performance of 127 µg/√Hz.

A torsional cantilever topology is used to implement the vibrational sensor, wherein the proof mass is supported by torsional tethers at one end (Fig. 1b, c). Micro-gravity level accelerations applied due to acoustic vibrations of the chest wall generate a torque, which in turn rotates the proof mass, resulting in change in capacitance at the sense electrodes. The sense electrodes are placed within the proof mass as shown in Fig. 1b to maintain a small form-factor, and a differential top electrode configuration is employed to suppress common mode noises. The sensors are designed to be low-noise (<10 µg/√Hz), and have high bandwidth (f_res_ > 10 kHz), thus providing high sensitivity to micro-gravity level accelerations orthogonal to the device surface.^35^

To overcome the tradeoff between low-noise performance and extended bandwidth, the capacitive gap size is scaled down to ultra-thin nanogaps, which enable a high operational frequency without compromising on device size and sensitivity. The device is fabricated using the HARPSS+ process on a silicon-on-insulator wafer having 40 μm thick device layer with 270 nm capacitive gaps as shown in Fig. 1a. Fabricated devices are then wafer-level packaged using eutectic bonding to a silicon capping wafer with built-in through-silicon-vias (TSV) shown in Supplementary Fig. 1a. The capping wafer ensures a low-pressure environment (10 Torr) to minimize effects of squeeze-film damping during device operation. Additionally, the wafer-level packaging safeguards the microdevice from environmental and accidental damage. Figure 1d shows a sensitivity of 76 mV/g and an extremely linear response under high acceleration. Cross-axis sensitivity of less than 3% is measured, occurring mainly due to misalignments of the sensor to the measurement system rather than device design. Figure 1e demonstrates the low-noise performance of the microsensor, exhibiting a velocity random walk of 127 µg/√Hz, which is limited by the interfacing electronics. The Brownian noise floor of the MEMS device is <10 µg/√Hz making it an ideal candidate for sensing mechano-acoustic signals. Supplementary Fig. 1c displays the measured resonant frequency of 12.5 kHz when the device is placed under vacuum conditions, confirming a wide operational bandwidth. The specifications of the sensor are listed in Supplementary Table 1.

The fabricated sensor is packaged and housed within a wearable chest band such that the device is in direct contact with the user’s thoracic region to monitor vital health parameters. The sensor’s wide dynamic range prevents output saturation during ambulatory conditions, while being highly sensitive to micromovements produced by the body during respiration and cardiovascular activity. The sensor, being a quasi-static device, is responsive to vibrations ranging from low frequency levels to audible frequencies, which enables acquisition of high fidelity SCG signals from the heart as well as monitoring of respiratory rate and breathing patterns of the user that occur in the infrasonic range (<20 Hz). The sensor unifies the functionality of an inertial device, accelerometer, used for measuring activity and body motion, with an auscultatory sensor such as a microphone for capturing heart and lung sounds, into an integrated sensor platform. The encapsulated microsensor is thereby termed an Accelerometer Contact Microphone (ACM).

### Recording cardiopulmonary sounds and body motion

Various physiological acoustic signatures originate from our body, occurring over a wide range of frequencies. Applications of SCG correspond to low frequency signals (<20 Hz),^16^ while the heart sounds are present at a higher frequency range of 20–200 Hz.^36^ Additionally, the respiratory sounds are much weaker signals occurring in a wider frequency range of 60–6000 Hz.^17^

**Fig. 2:**
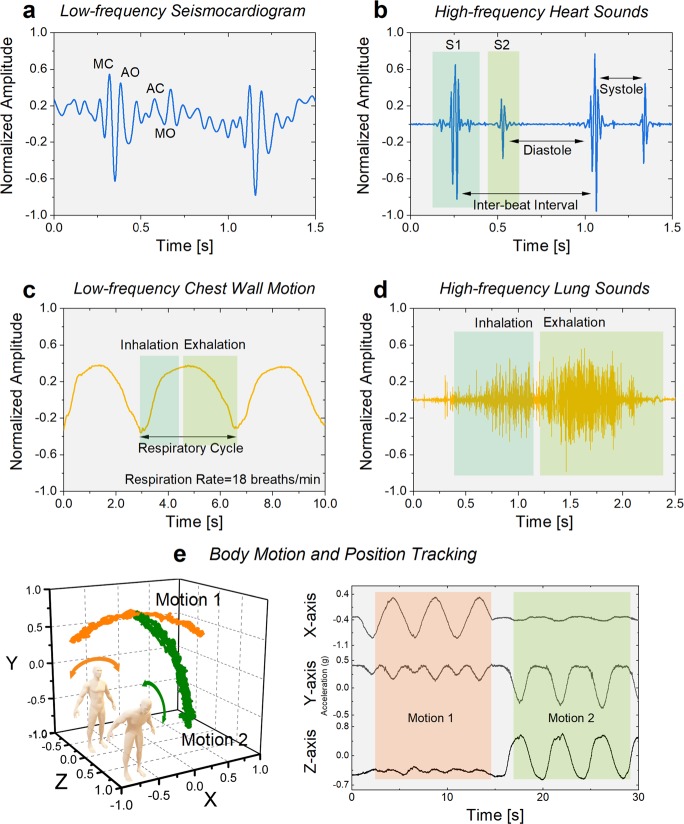
Recording cardiopulmonary vibrations, sounds and body motion. **a** Time domain plot of measured SCG signal. Peaks corresponding to occurrence of closing of mitral valve (MC), opening of aortic valve (AO), closing of aortic valve (AC) and opening of mitral valve (MO) are indicated. **b** Recorded waveforms of two cardiac cycles showcasing sensitivity to the two major cardiac sounds (S_1_ and S_2_). Time intervals of inter-beat, systole and diastole are specified. **c** Sensor output signal representing motion of the chest wall during deep-breathing respiratory cycles. Time intervals of inhalation and exhalation are identified for computation of respiratory rate. **d** High frequency lung sounds of inhalation and exhalation as recorded by the vibration microsensor. **e** Body motion tracking in three dimensions, using the ACM along with two in-plane accelerometers, as the individual performs side-to-side (orange) and frontal (green) bending exercises. Time domain plots recorded during exercising showcasing the wide dynamic range of the sensor.

The SCG signal, defined as the micromovements of the chest wall in response to pumping of blood at every heartbeat, is recorded as shown in Fig. 2a in the inaudible frequency band and its importance is signified by its unobtrusive nature and ability to detect early onset of congestive heart failure as well as acute myocardial disease and asymptotic coronary artery disease.^37,38^ The waveforms comprise peaks corresponding to occurrence of closing of mitral valve (MC), opening of aortic valve (AO), closing of aortic valve (AC), and opening of mitral valve (MO). The two major cardiac sounds, S_1_ and S_2_, which occur due to closing of the atrioventricular valves (mitral and tricuspid) and closing of the semilunar valves (aortic and pulmonary), respectively, are recorded as shown in Fig. 2b. The systolic and diastolic time periods can be used for automatic heart sound classification and diagnosis. The frequency content and timing of the recorded heart sounds can be used for identification of abnormal heart sounds such as murmurs which may occur due to turbulent blood flow.^36^ The performance of the sensors in terms of recording cardiopulmonary sounds is validated with a medical-grade electronic stethoscope (see Supplementary Fig. 2). The waveforms of concurrently recorded signals from the two methods are compared, and the results demonstrate a strong correlation of 70% similarity in the time domain signals of the heart sounds. This variation in similarity occurs mainly due to the difference in sensitivity of the ACM and the electronic stethoscope, as well as minor deviations in sensor position when placed adjacently on the user’s chest. We also observe that the high signal-to-noise ratio of the ACM may facilitate improved detection of S_2_ heart sound occurring in the higher frequencies (~140 Hz).

Respiratory sounds provide vital information regarding pulmonary health such as elucidating obstructions in airway or presence of liquid in the organ. By monitoring the chest wall motion of the individual, we can measure the respiratory rate and breathing patterns to potentially predict early onset of chronic cardiopulmonary conditions. Figure 2c demonstrates the periodicity of the breathing pattern and identification of respiratory phases within the waveform in a healthy individual (See Supplementary Fig. 9 for additional breathing patterns). The characteristics of respiratory sounds are modulated as air passes through the lungs, resulting in varied pitch and duration of recorded lung sounds with respect to location of the senor. While healthy subjects exhibit vesicular breath sounds occurring around the 600 Hz frequency range (Fig. 2d), patients suffering from congestive heart failure and pleural effusion often present a crackle, indicating accumulation of fluid within the lungs. The durations of inhalation and exhalation coupled with the recorded lung sounds during the respiratory cycle hold key information regarding the physiological functioning of the organ.^17,39^

Integration of health monitoring biosensors with regular inertial devices is challenging due to the limited dynamic range of sensors, resulting in either saturation of the biosensors or inability of inertial sensors to capture biomechanical signals. The high dynamic range of the ACM allows it to simultaneously monitor both—cardiopulmonary sounds and body motion—without saturation. This functionality of the ACM is demonstrated in Fig. 2e, using an enhanced ACM platform comprising two additional high-precision in-plane accelerometers for complete three-dimensional body motion and position tracking. The subject performs two activities—side-to-side bending followed by frontal bending—which simulate the range of motion during regular activities. The resultant ACM response is shown alongside, clearly identifying each body motion individually.

### Computed health parameters

**Fig. 3:**
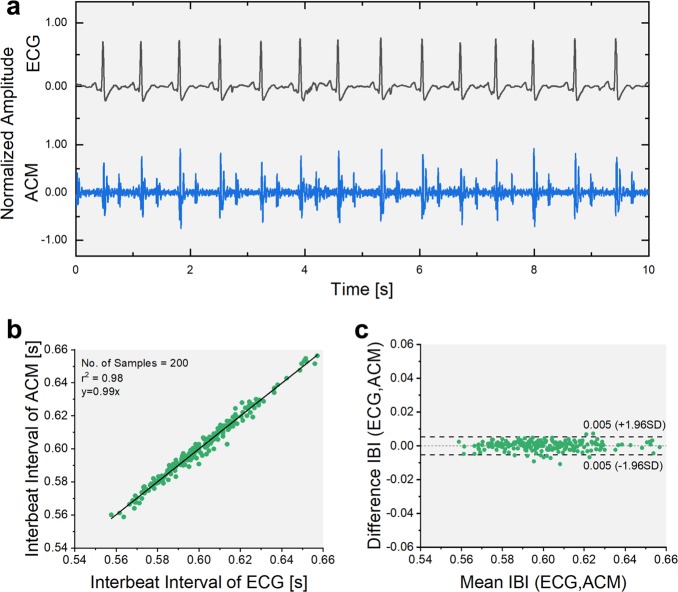
Comparison and computation of diagnostic parameters. **a** Time domain plot of measured ECG signal alongside the ACM. **b** Correlation plot illustrating linear curve fit with r^2^ = 0.98 confirming effective use of ACM for computation of diagnostic parameters. **c** Bland-Altman plot comparing the technologies demonstrates 95% confidence interval having a range of 0.01 s.

The sensor is applied to compute health parameters such as heart rate variability (HRV) and inter-beat intervals (IBIs) by extracting the time intervals based on the vibrational signatures of cardiac activities. Figure 3a displays the simultaneously recorded time domain signals of a medical-grade ECG alongside the ACM. Analogous to the R-peak of the ECG signal (Supplementary Fig. 3a), the opening of the aortic valve is used as a reference peak to segment the recorded signal into individual beat components and obtaining an ensemble averaged signal (Supplementary Fig. 3b). The IBIs calculated using the two methods demonstrates a high correlation, indicating a linear fit with r^2^ = 0.98 (Fig. 3b). A Bland-Altman plot quantifies this similarity showing the 95% confidence interval having a range of 0.01 s (Fig. 3c). This signifies the use of the ACM for vital parameter monitoring in place of ECG electrodes without compromising on signal strength, quality and accuracy, while providing additional benefits of auscultatory ability simultaneously. The microsensor was tested on six control subjects to validate its operability over varied body types. The HRV, inter-beat, systolic, and diastolic time intervals are computed for these six healthy subjects as shown in Supplementary Table 2. Supplementary Fig. 3c displays the ensemble averaged time domain signals recorded from the six healthy control subjects.

### Detection of weak mechano-acoustic signals

**Fig. 4:**
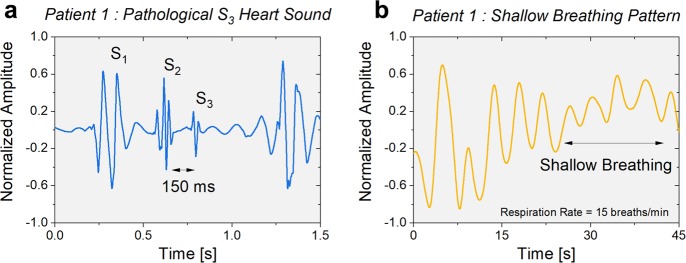
Validating detection of weak mechano-acoustic signals. **a** Time domain plot of measured cardiac sounds. Presence of abnormal ventricular gallop (S_3_) occurring 150 ms after S_2_ heart sound. **b** Breathing pattern monitored by the ACM shows instances of shallow breathing indicating shortness of breath. Respiratory rate of 15 breaths per minute is calculated based on periodicity of the signal.

Preliminary evaluation of the vibration sensor is conducted on three patients with preexisting cardiopulmonary conditions. The patients had wide ranging BMI (18.8–41) and were in the age group of above 40 years old and were all males. A protocol involving one-minute rest period followed by 6 min of exercise and 2 min of rest is employed. Recorded signal from a single sensor is used to provide the multi-faceted health analysis concerning heart sounds, respiratory rate, HRV, IBI, and body motion. Figure 4a displays the recorded pathological ventricular gallop, often referred as *S*_*3*_ heart sound, occurring about 150 ms after the *S*_*2*_ (see Supplementary Fig. 4 for waveforms corresponding to presence of S_3_ heart sound in remaining patients). The *S*_*3*_ sound is an important early diagnostic marker for patients with low cardiac output resulting from congestive heart failure.^20,21,40^ A respiratory rate of 15 breaths per minute with a shallow breathing pattern during rest period is recorded suggesting shortness of breath as shown in Fig. 4b. Supplementary Fig. 4a demonstrates the activity chart for the patient showing the exercise phase followed by a rest period. The two peaks within the rest period time interval occur due to adjustments in the posture and position of the patient. The computed diagnostic parameters are presented in Supplementary Fig. 4b showing a low heart rate variability of 15 ms and an IBI of 1.05 s. These markers provide critical information for assessing the individual’s health and assist in managing cardiopulmonary conditions.

## Discussion

Early detection of pathological signatures in mechano-acoustic signals is critical to potentially delay the progression of cardiopulmonary diseases and improve the quality of care. Our wearable vibration sensor detects abnormal sounds and symptoms of cardiopulmonary diseases in early stages and provides a unique solution for disease screening and longitudinal monitoring. The sensor, being responsive to a wideband of vibrational frequencies and exhibiting a high dynamic range, senses micromovements with high precision along with the macro body motion of the individual, adding an additional layer of information for correlation of auscultation to daily activities. The sensor, being a contact microphone, only responds to vibrations from its contact surface while being insensitive to airborne acoustic emissions (experimental results shown in Supplementary Fig. 6), making it suitable for use in wearable applications. High frequency components recorded in the audible range constitute the cardiopulmonary sounds produced within the thoracic cavity, while the low frequency inaudible content comprise the SCG and body motion signals. Due to overlap in frequency ranges for certain applications such as SCG and body motion, advanced signal processing techniques are needed to separate the signals. One effective technique is to record the signals for a long duration of time and use methods of ensemble averaging to improve the signal-to-noise ratio (as shown in Supplementary Fig. 7). Alternatively, multiple ACM sensors can be used simultaneously to record signals and applying processing techniques such as decomposition,^41^ independent component analysis,^42^ and modulation filtering^43^ to reduce motion artifacts. These features of sensing the SCG, cardiopulmonary sounds, breathing pattern, and body motion, in conjunction with computed health parameters of HRV and IBI offer exclusive benefits of multi-dimensional health monitoring that are currently unavailable. The current piezoelectric sensors with limited lower operational frequency of 20 Hz are unable to capture clinically relevant data concerning the SCGs and breathing patterns, which are known parameters for early diagnosis and evaluation of cardiac dysfunctions. To ensure a holistic diagnosis, it is imperative to capture mechanical motion of the heart and generated cardiopulmonary sounds, which can be acquired using the presented device, along with the ECG signal to enhance diagnostic accuracy.

In summary, we demonstrated that heart rate, heart sounds, respiratory rate, lung sounds, and body motion of an individual can be simultaneously recorded in a continuous and unobtrusive manner using a single integrated sensor. Proof-of-concept studies were conducted on healthy control subjects as well as patients with preexisting conditions to monitor physiological and pathological mechano-acoustic signals emanating from the heart and lungs. This opens the possibility of identification, early detection and long-term monitoring of cardiopulmonary dysfunctions.

## Methods

### ACM fabrication process

The ACM is fabricated using a combination of CVD and RIE, with the nanogaps defined by the thermal oxidation of silicon,^34^ as shown in Supplementary Fig. 1. A (100) silicon-on insulator (SOI) wafer with 40 µm device layer is used as the base layer, which is thermally oxidized to form a hard mask. The oxidized layer is patterned, and using DRIE techniques, trenches are etched in the device layer. The DRIE trenches are then filled using LPCVD tetraethyl orthosilicate (TEOS) through a process of repeated conformal deposition and top surface etch back. The top surface is then patterned, which exposes the regions for transduction and electrode anchoring. The exposed region is thermally oxidized to form the top sacrificial oxide layer (270 nm in thickness) for the sense electrodes. Finally, polysilicon is deposited and patterned to for the sense electrode. The wafer is released in HF solution using a super critical point drier. The capping wafer is on a silicon wafer. Through-silicon vias (TSV) are formed using deep polysilicon pillars with oxide isolation. These are used to create an external connection path from the capping wafer to the sense electrodes. A deep-cavity is etched using DRIE, whose depth is engineered to control the package pressure level. The capping wafer is then bonded using high-vacuum eutectic bonding. Finally, the capping wafer is grinded to expose the TSV, which is followed PECVD oxide and metal electroplating to form the electrical routing on the packaged device.

### System development and signal processing

The capacitive transducer is wire-bonded to an electronic board with the size of 2 × 2 cm (off-the-shelf electronics—MS3110 by Irvine Sensors—on the other side of the board to read capacitive change). To measure sensitivity, the board is mounted on the shaker table and a sinusoidal 1 g acceleration is applied at 1 kHz frequency. Dynamic Signal Analyzer 35760 A is used to precisely measure the scale factor, showing 72.6 mV/g. The resonant frequency of the device was measured by placing uncapped device into the vacuum chamber and exciting electrostatically using network analyzer E5061B. The measured resonant frequency is 12.5 kHz, confirming high operational bandwidth.

A chest band prototype is developed to enable use of the ACM in a wearable form-factor. Elastic bands are used to ensure a snug fit over the user, and it enables adequate contact pressure while suppressing the undesired rubbing noise that may arise due to motion. The sensor is sandwiched between two layers of 4-inch wide elastic bands with a slit to expose the contact surface for the device. Hook-and-loop fasteners are used at the ends for securing the chest band around the user. The band is worn around the chest such that the sensor is placed directly over the sternum for maximum output signal. Only the sensor area is in contact with skin and this device did not cause any burden for the wearers. A data acquisition system with sampling rate of 8 k samples/sec is used to transfer analogue data to computer. Data processing and filtering techniques are employed using a MATLAB program to separate the low frequency component of the chest wall motion from the high frequency audible components. The SCG signals correspond to micromovements of the body at low frequencies (<20 Hz), while the heart sounds occur at a higher frequency range of 20–200 Hz. Due to the non-overlapping nature of these two signals, an IIR low pass filter (20 Hz) and band pass filter (20–200 Hz) is applied to remove the undesired noise. A wavelet denoising algorithm is applied to further reduce the noise artifacts and obtain a higher signal-to-noise ratio, as shown in Supplementary Fig. 8. To perform segmentation of the signal, the indices of the S_1_ peaks is obtained by normalizing the signal and finding peaks over certain threshold. Distance between two peaks is limited to avoid misidentification of an S_2_ peak as an S_1_ peak. Using the index locations of the peaks, the original ACM signal is segmented into individual beats.

### Pilot study testing

All the human subjects participate voluntary with informed consent. The protocol was approved by Emory University and Georgia Institute of Technology Institutional Review Board (IRB# H18248). The process of data collection from patients was supervised by an experienced and authorized cardiologist, and the sensors were attached to patients by cardiologists and nursing staff in Emory Heart and Vascular Center at Emory University Hospital.

### Reporting summary

Further information on research design is available in the Nature Research Reporting Summary linked to this article.

## Supplementary information

Supplementary Information

Reporting Summary

## Data Availability

All recorded datasets will be available to any investigator upon request.

## Code availability

The code will be available to any investigator upon request.
