# Supplementary material for: Precision wearable accelerometer contact microphones for longitudinal monitoring of mechano-acoustic cardiopulmonary signals
Source: NPJ Digit Med. 2020 Feb 12;3:19. doi: 10.1038/s41746-020-0225-7 (PMC7015926; doi:10.1038/s41746-020-0225-7)
Supplement: Supplementary file 1 — Supplementary Information [file 41746_2020_225_MOESM1_ESM.pdf]

Supplementary Notes

Auscultation in Noisy Environment

Absence of auscultation in telemedicine and remote health monitoring platforms can be attributed to a dearth of wearable continuous monitoring devices capable of capturing body sounds with little or no distortion while being unaffected by external environmental noise. State-of-art digital stethoscopes employ active noise cancellation technologies to achieve such performance, resulting in bulky devices with high power consumption which are unsuitable for use in ambulatory systems and can be only used in bed-side or clinical settings. The ACM offers an exciting new solution to tackle these challenges by exploiting the vibration sensor's intrinsic ability to capture sounds from its contact surface while being unresponsive to environmental noise, and hence create a low-power wearable device.

To evaluate the performance of the ACM in a noisy environment, the ACM is placed on the chest of the user along with an airborne microphone (Knowles SPW2430). A speaker system is placed next to the subject to simulate daily environmental noise with adequate loudness, as shown in supplementary Fig.6. Data from both sensors are recorded simultaneously and waveform features are compared in time domain. Inspecting the acquired waveforms, we observe that upon increasing the airborne noise, we notice no significant changes to the ACM signal quality. Based on the output signal levels of the airborne microphone, the maximum airborne noise level is 103.5db SPL. During this experiment, the airborne noise level is limited by the speaker output. This validates the use of ACM in extremely noisy environments without any degradation in signal quality.

To validate the performance of the ACM to acquire clinically relevant information, we compare its performance against a medical-grade electronic stethoscope, as shown in supplementary Fig.2. The stethoscope chest piece and the ACM are placed adjacently on the

chest of the subject. Time domain signals from both sensors are recorded, segmented and denoised to obtain a high-fidelity heart sound signal. A Pearson correlation coefficient analysis indicates a similarity of 70% between the two signals. This variation in similarity occurs due to the difference in sensitivity of the ACM and electronic stethoscope, as well as deviation in sensor position when placed on the user's chest. To further investigate the sensing accuracy, we plot the power spectrum of the two signals in the frequency range of 10 Hz – 200 Hz. This frequency range corresponds to the major concentration of energy of the two heart sounds (S1 and S2). We observe that the ACM and the electronic stethoscope have a high correlation signified by an overlapping power spectrum in supplementary Fig. 2(d) at low frequencies (below 100Hz) corresponding to the S1 heart sound. As we move higher in the frequency spectra, the higher sensitivity of the ACM enables detection of weaker S2 heart sounds, and we observe a peak at ~140Hz for the ACM signal. This difference in sensitivity to weak S2 heart sounds is also visible in the time domain signal of the two sensors, thus validating the detection of clinically relevant cardiopulmonary sounds with high fidelity."

Supplementary Figures

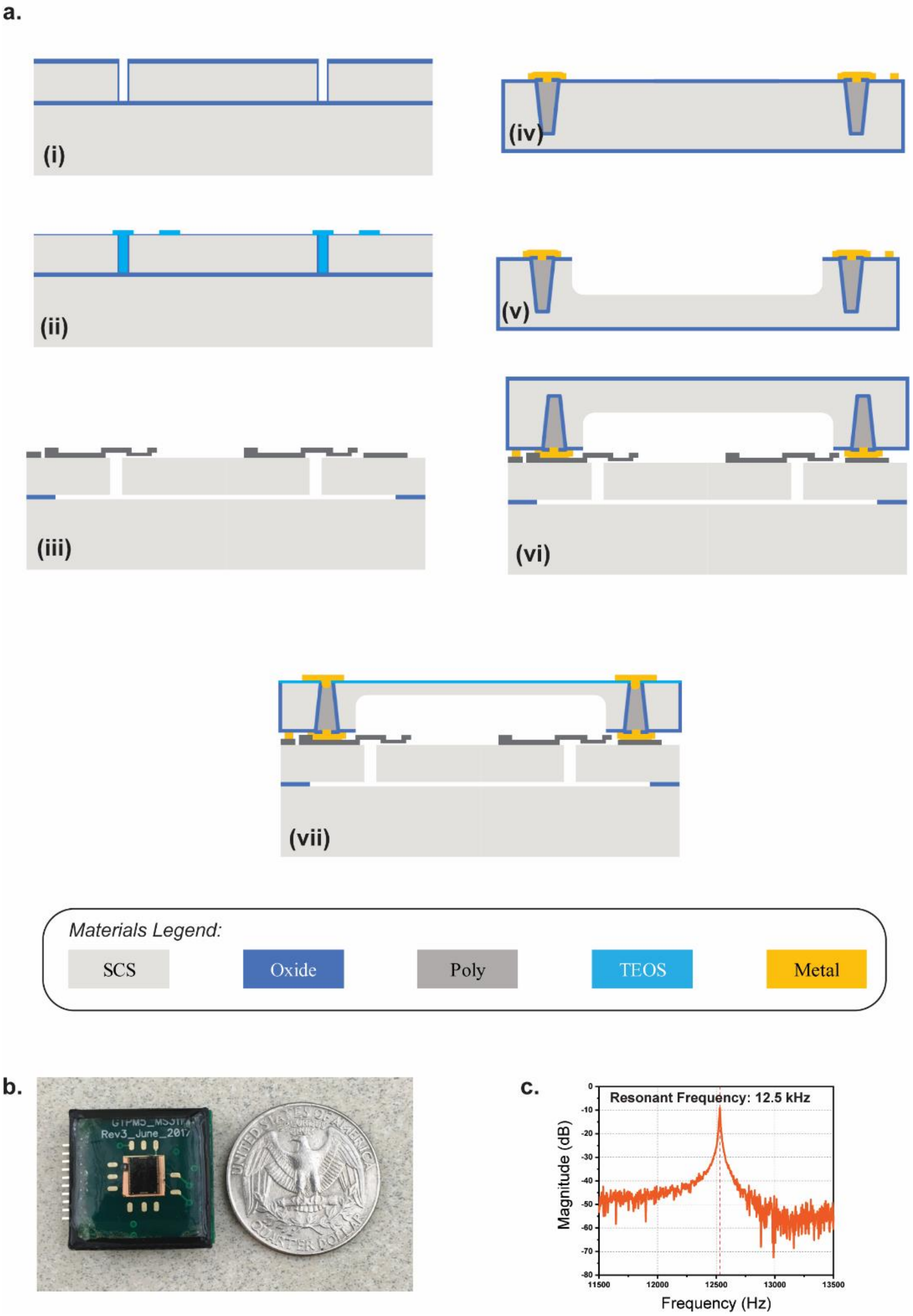

Supplementary Figure 1 | a. Cross-sectional view illustrating step-by-step fabrication of the ACM. (i) (100) silicon on insulator (SOI) wafer with 40µm device layer is used as the base layer. Trenches are etched using DRIE in the device layer. (ii) The DRIE trenches are then filled using LPCVD tetraethyl orthosilicate (TEOS). The exposed region is thermally oxidized to form the top

sacrificial oxide layer (270nm in thickness) for the sense electrodes. (iii) Polysilicon is deposited and patterned to for the sense electrode. The wafer is released in HF solution using a super critical point drier. (iv) The capping wafer is on a silicon wafer. Through-silicon vias (TSV) are formed using deep polysilicon pillars with oxide isolation. (v) A deep-cavity is etched using DRIE, whose depth is engineered to control the package pressure level. (vi) The capping wafer is then bonded using high-vacuum eutectic bonding. (vii) The capping wafer is grinded to expose the TSV, which is followed PECVD oxide and metal electroplating to form the electrical routing on the packaged device. **b**. vibration sensor interfaced with readout electronics on a miniature circuit board (2cm x2cm) with protective epoxy coating. (coin shown for size comparison) **c.** Measured resonance frequency of 12.5kHz of the microsensor under vacuum conditions.

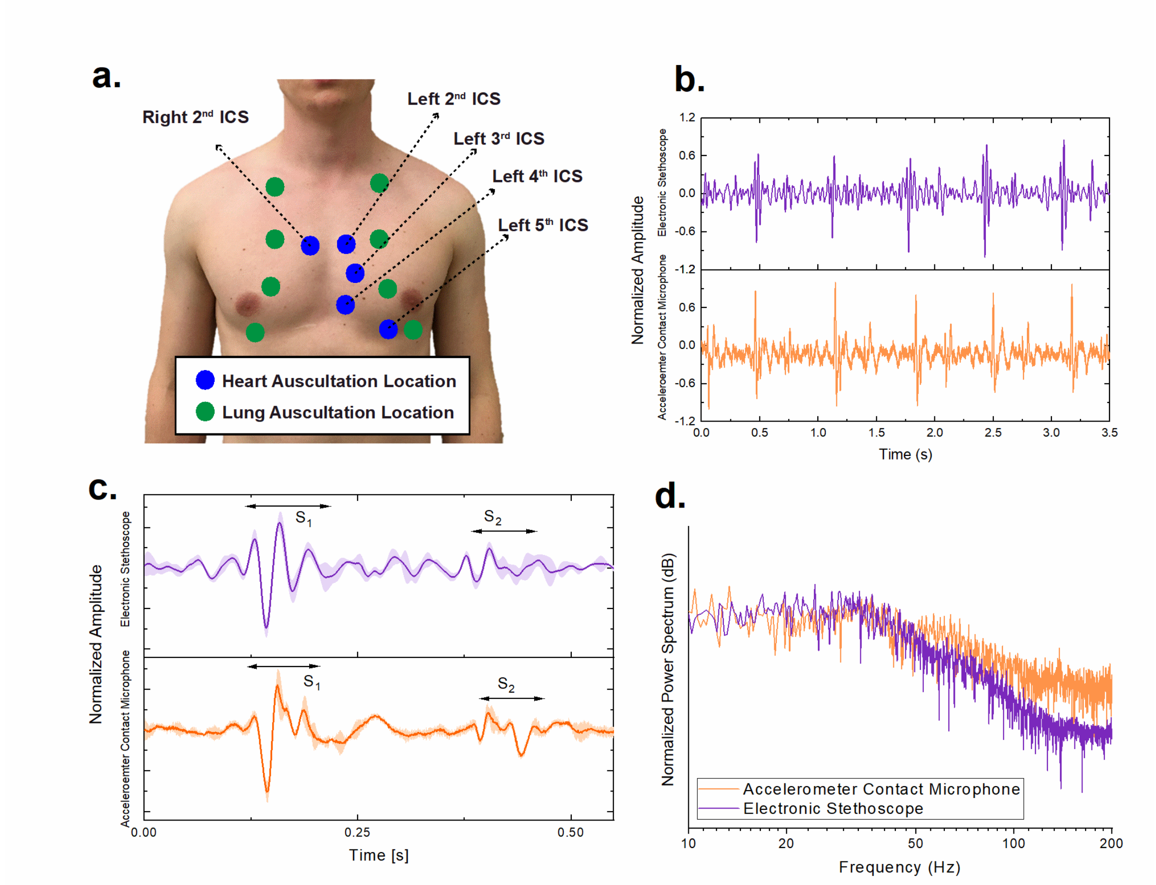

Supplementary Figure 2 | Auscultation locations and comparison with stethoscope. a. Several locations for cardiopulmonary sounds in the intercostal spaces (i.e. the space between two consecutive ribs) on the chest, **b.** comparison of simultaneously recorded time domain signals of a medical-grade electronic stethoscope and ACM **c.** Ensemble averaged time domain signals of the ACM and stethoscope. The Pearson correlation coefficient indicates a strong similarity of 70% in the two signals. **d.** comparison of power spectrum of the recorded signals demonstrating the ability of the ACM to capture heart sounds with high-fidelity, comparable to the electronic stethoscope.

Written informed consent was obtained for using the image of the person in the above figure.

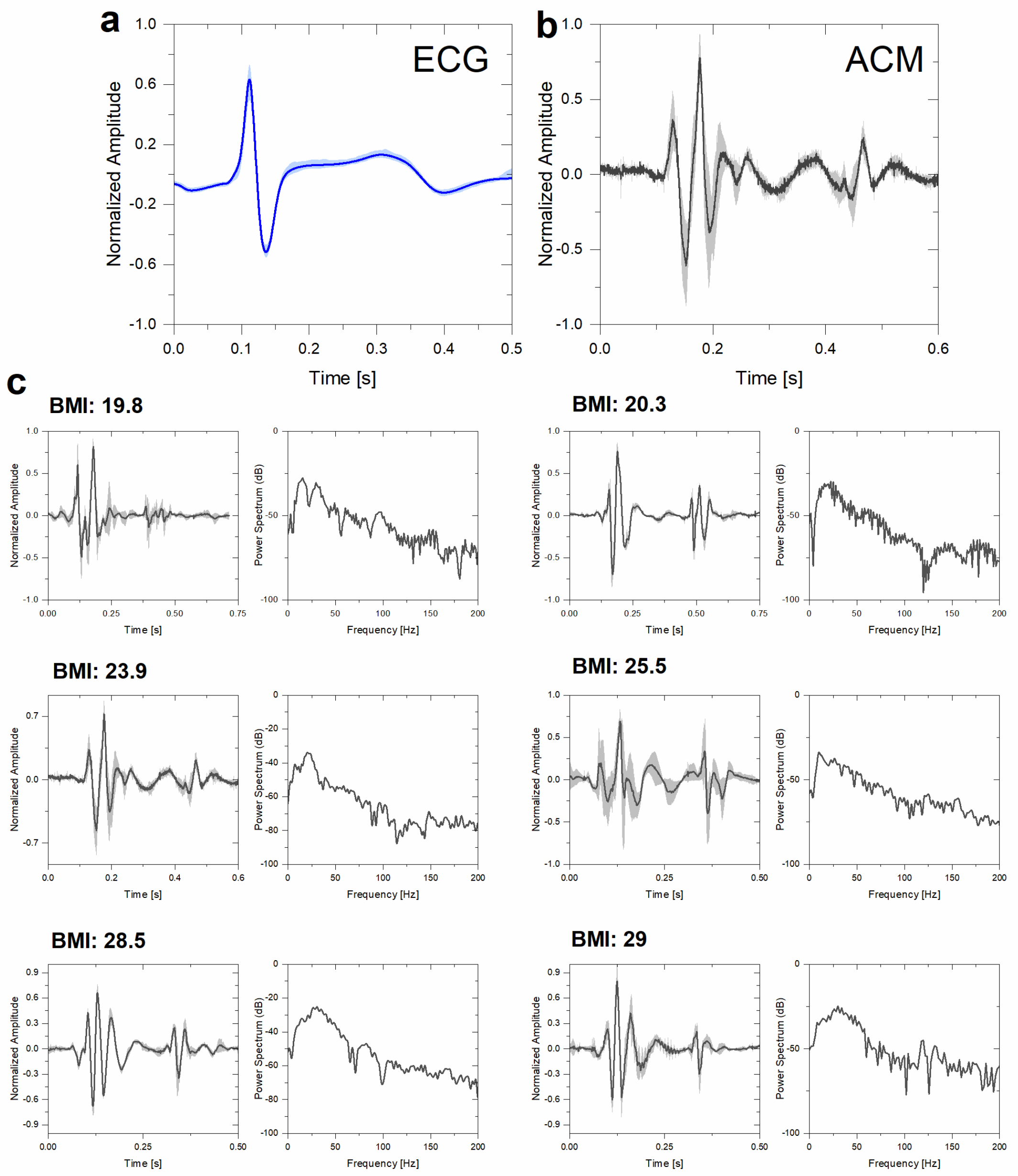

Supplementary Figure 3 | Comparison of ACM time domain signals. a. ECG signal recorded using Biopac MP150 System. **b.** ACM time domain signal recorded simultaneously alongside the ACM. **c.** ACM time domain signals across varying body mass index (BMI). Frequency plot demonstrates the maximum signal power.

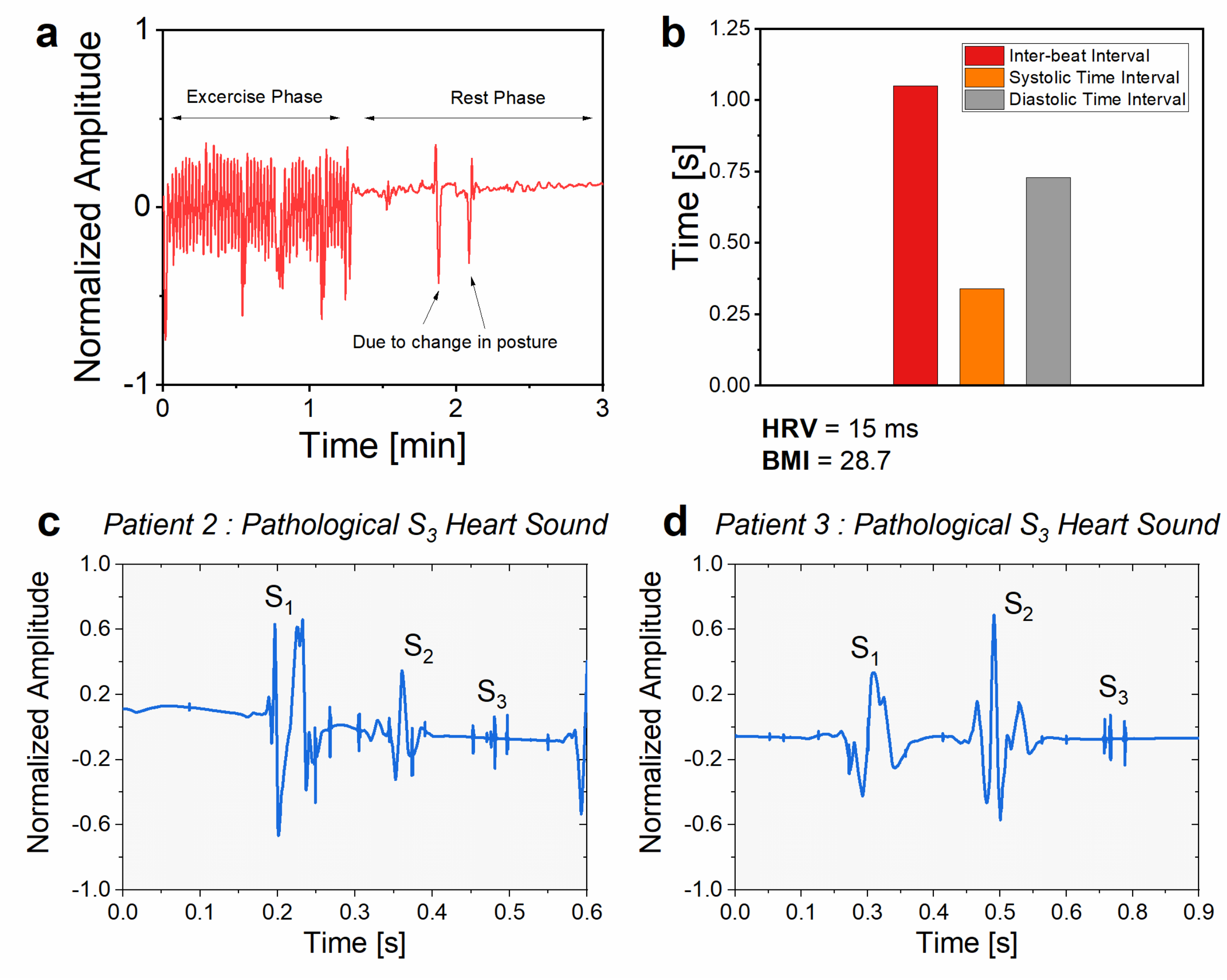

Supplementary Figure 4 | Data from patients with preexisting cardiopulmonary conditions a. Body motion and position analysis of patient 1. **b.** Computed diagnostic parameters of Patient 1. **c.** Presence of pathological S_3 heart sound in male patient with BMI 18.8 and ejection fraction (EF) 15%. **d.** Presence of pathological S_3 heart sound in male patient with BMI 41 and EF 5%.

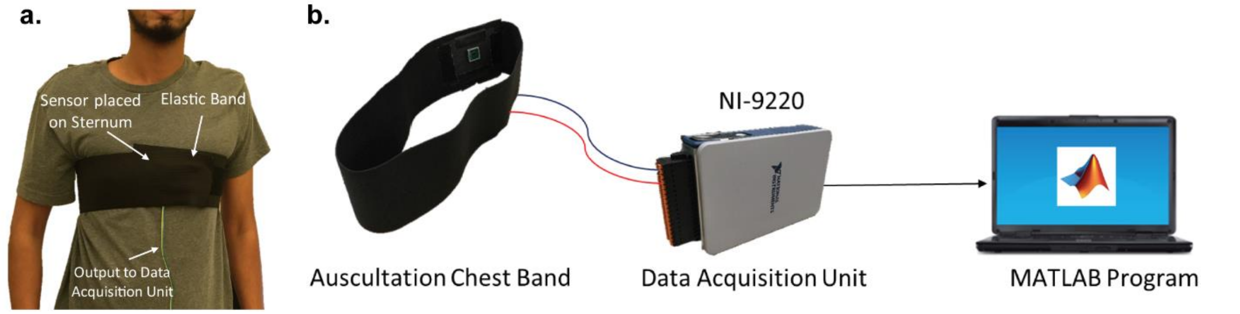

Supplementary Figure 5 | Wearable chest band with vibration sensor. a. Elastic chest band housing the vibration sensor is worn over T-shirt such that the sensor is placed at the sternum. This output voltage from the device is fed into a data acquisition unit using cables. **b.** Schematic system level diagram illustrating the implemented system design utilizing a data acquisition platform (NI-9220) along with a MATLAB program.

Written informed consent was obtained for using the image of the person in the above figure.

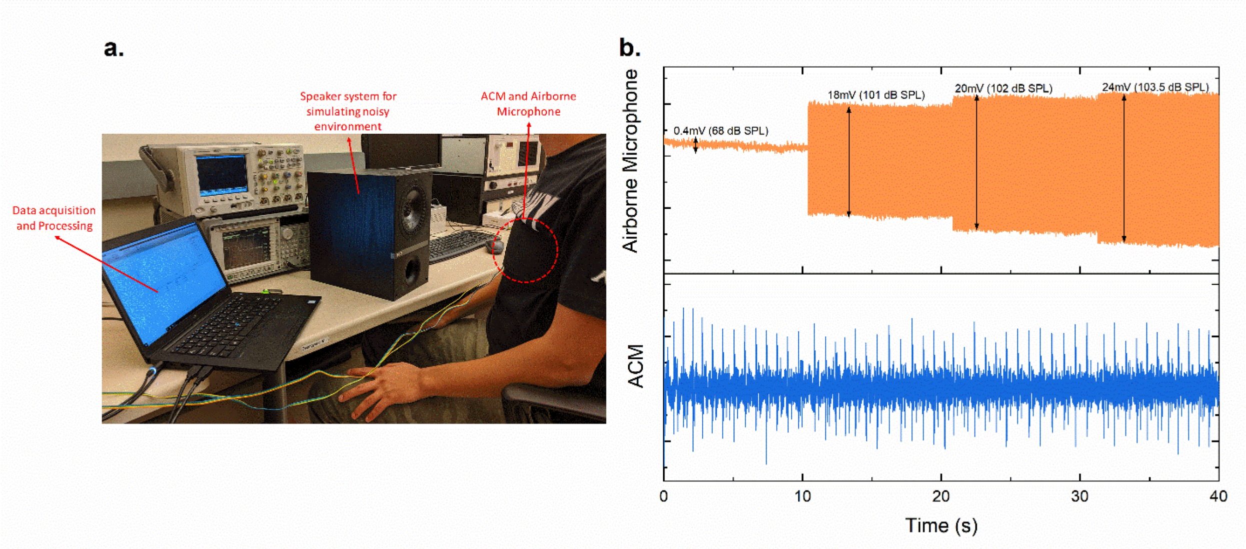

Supplementary Figure 6 | Demonstration of auscultation in a noisy environment. a. experimental setup showcasing the ACM placed alongside an airborne microphone. A speaker system is used to simulate the noisy environment. **b.** Raw data of the time domain signals recorded using the two sensors. The ACM signal quality does not deteriorate with increased noise levels.

Written informed consent was obtained for using the image of the person in the above figure.

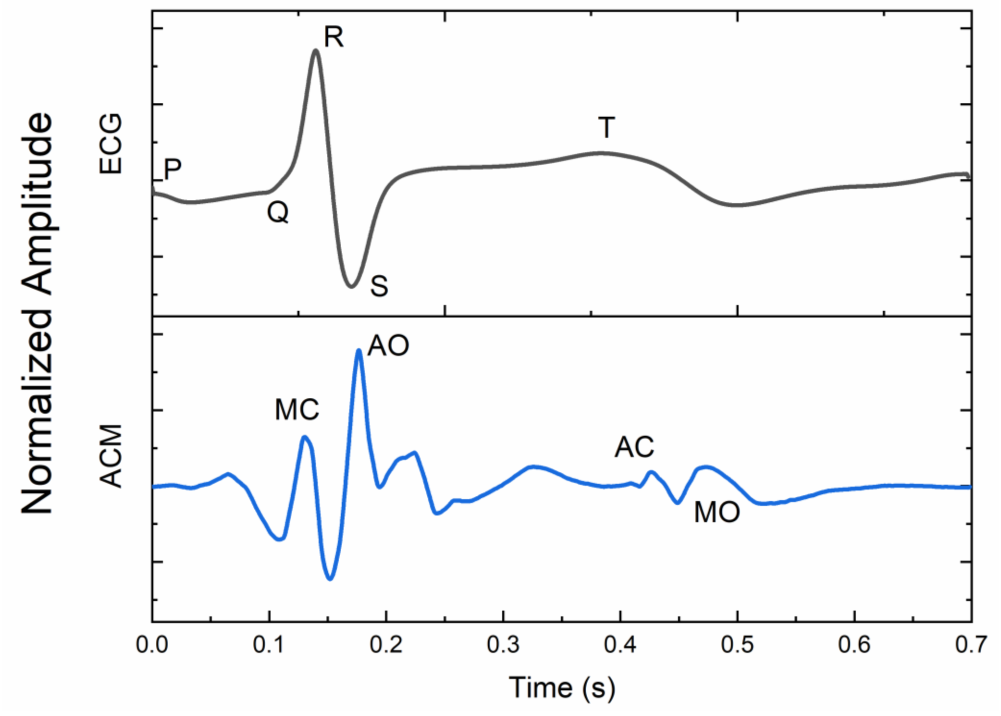

Supplementary Figure 7 | Comparison of SCG and ECG waveforms. ECG signal is recorded using a 3-lead monitor, and the SCG signal is captured using the ACM. The two signals were simultaneously recorded under stable, stationary conditions. Ensemble-averaging is applied to improve the signal-to-noise ratio of the SCG signal. The graph highlights the significant peaks and their corresponding representative functions (MC,AO,AC,MO).

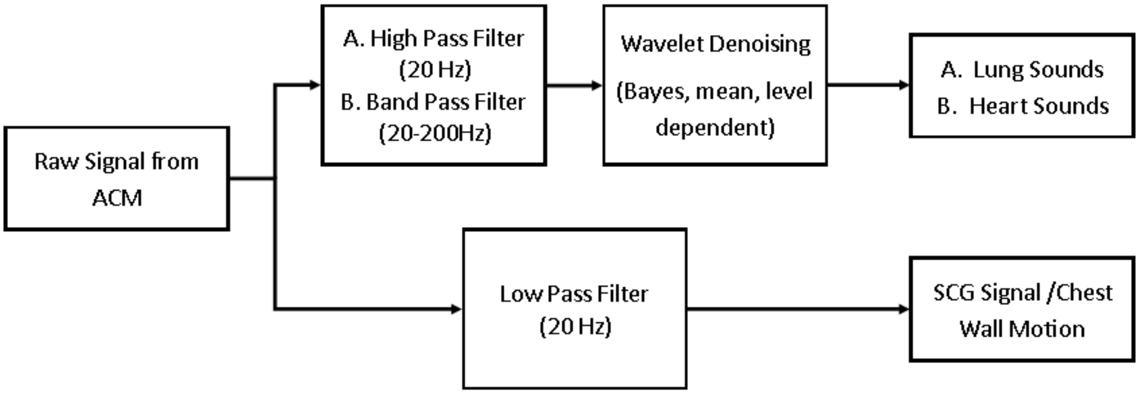

Supplementary Figure 8 | Signal Processing Flow. The raw ACM signal is filtered using a low pass IIR filter (20Hz) to obtain the low frequency body motion and SCG signal. Band pass filtering (20-200Hz), followed by wavelet denoising is applied to obtain high-fidelity heart sounds.

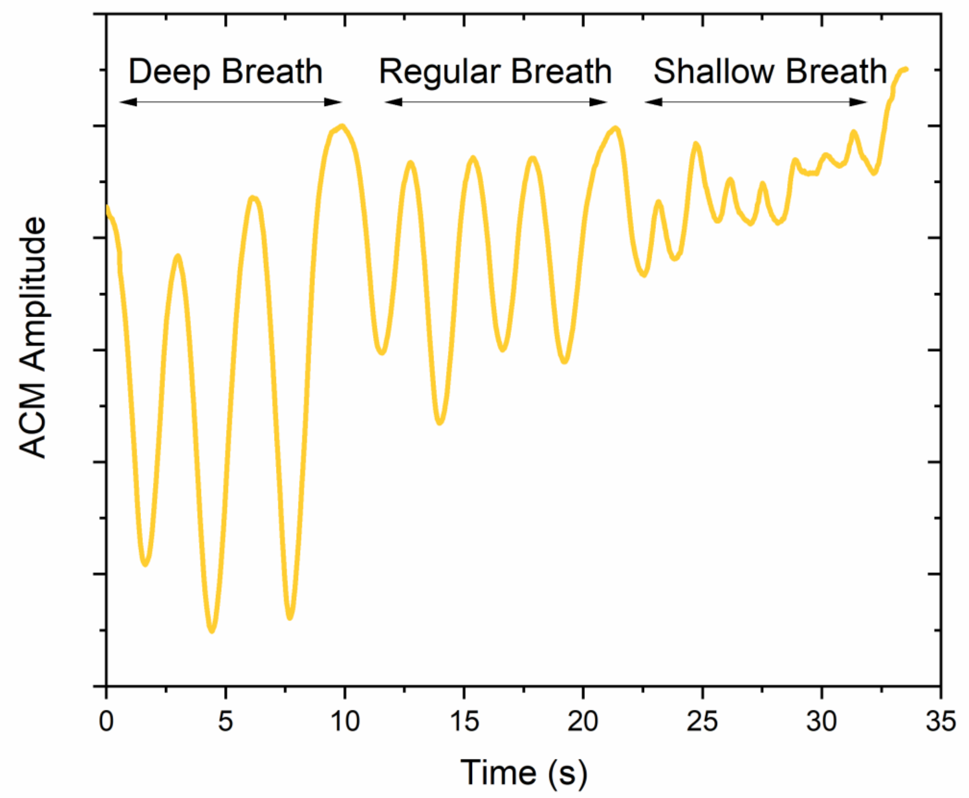

Supplementary Figure 9 | Different breathing patterns. Various breathing patterns corresponding to deep breaths, regular breaths and shallow breaths recorded using the ACM. These breaths are recorded from a healthy person and only represent the expected relative amplitude and pattern. The amplitude of these breathing patterns differ from person to person as well as across the sensor locations.

Supplementary Table 1. Specifications for the Accelerometer Contact Microphone

Parameter	Value
Sensitivity	76 mV/g
Resonant frequency	12.5 kHz
Linear dynamic range	160 m/s^2 (16 g)
Die size	2 mm×2 mm×1 mm
Gap size	270 nm
Measured Noise density	127 μg/√Hz (@ 1 Hz)
Brownian Noise of MEMS	3.6 μg/√Hz (@10 Torr Pressure)
Measured Cross axis sensitivity	<3%

Supplementary Table 2. Computed Health Parameters for Control Subjects

Subject ID	Body Mass Index	HRV [ms]	Interbeat Interval [s]	Systolic Time Interval [s]	Diastolic Time Interval [s]
1	20.3	25	0.83	0.32	0.55
2	25.5	31	0.72	0.26	0.42
3	19.8	61	0.79	0.3	0.5
4	23.9	11	0.61	0.25	0.35
5	29	49	0.76	0.32	0.42
6	28.5	15	0.61	0.26	0.34
